# A methodological framework for fractal analysis in monitoring periapical lesions after endodontic retreatment: a comprehensive parameter optimization study

**DOI:** 10.1186/s12903-026-09179-5

**Published:** 2026-07-16

**Authors:** Kader Azlağ Pekince, Adem Pekince, Yağız Özbay

**Affiliations:** 1 Department of Oral and Maxillofacial Radiology, Karabük University Faculty of Dentistry, Karabük, Türkiye; 2 Department of Endodontics, Karabük University Faculty of Dentistry, Karabük, Türkiye

**Keywords:** Fractal Analysis, Endodontic Retreatment, Periapical Lesion, ROI Selection, BoneJ Auto Algorithm, Gaussian Filter, Periapical Index, Bone Healing, Clinical Non-Healing Reference Group

## Abstract

**Background:**

This retrospective study aimed to investigate the efficacy of Fractal Analysis (FA) in the quantitative assessment of periapical bone healing after non-surgical endodontic retreatment, and to standardize the key methodological parameters — region of interest (ROI) selection, image processing filters, and box-counting algorithms — that govern its reliability.

**Methods:**

Ninety-five patients free of systemic conditions affecting bone metabolism who had undergone endodontic retreatment were divided into two groups: a healed group with PAI < 3 (*n* = 60) and a non-healed group with PAI ≥ 3 (*n* = 35). The non-healed group served as a clinical non-healing reference group to verify whether FA parameters remained stable in the absence of biological change. A total of 11,970 Fractal Dimension (FD) measurements were performed on 190 images (7 ROI types × 3 filters × 3 algorithms = 63 combinations). Seven ROI types (ROI-1 through ROI-7), three Gaussian filters (σ = 4, 12, 32 pixels), and three box-counting algorithms (B64, B32, BoneJ Auto [BA]) were tested. Mixed-effects GLM was used as the primary analysis; paired t-tests with Benjamini–Hochberg FDR correction (*q* < 0.05) served as the confirmatory analysis.

**Results:**

In the healed group, FD increased significantly over one year of follow-up (*p* < 0.001, Cohen's d = 1.07, AUC = 0.91 [ROI-1, BA + σ4; 95% CI: 0.85–0.96]). In the non-healed group, no significant FD change was observed for any combination (all *p* > 0.05, d ≤ 0.07). The highest discriminatory power was achieved with lesion-focused large ROIs, and the BA + σ4 combination yielded the strongest results across all significant regions.

**Conclusion:**

FA is a powerful quantitative tool complementary to PAI for monitoring endodontic healing. A lesion-focused circular/polygonal ROI (ROI-1/ROI-2), σ = 4 filter, and BoneJ Auto algorithm is recommended as a standardized protocol for 2D FA studies.

## Introductıon

Endodontic treatment is a fundamental dental intervention that aims to preserve dental integrity and function by eliminating the infected or damaged pulp tissue and hermetically obturating the root canal system with biocompatible materials [1]. Despite successful initial endodontic treatment, the prognosis can be adversely affected by various factors such as residual infections, inadequate canal disinfection, anatomical complexities, or operative errors, leading to treatment failure [2, 3]. These failures typically manifest as persistent or recurrent radiolucent lesions in the periapical bone tissue [4, 5]. In advanced cases, chronic inflammatory lesions may evolve into cyst-like pathologies, resulting in extensive alveolar bone loss, chronicity of symptoms, and eventual tooth extraction [6, 7]. Accordingly, non-surgical endodontic retreatment stands out as a vital treatment strategy for eradicating infection and promoting bone healing [3].

Accurate, objective, and quantitative assessment of periapical healing following endodontic treatment or retreatment is critically important for determining clinical prognosis and optimizing treatment strategies [3, 4]. The Periapical Index (PAI), defined by Ørstavik et al. (1986), is widely used in conventional radiographic evaluation [2, 8]. However, the observer-dependent, subjective nature of PAI and its limitations in discriminating subtle changes in periradicular bone carry the risk of missing early signs of healing [4, 7]. To overcome these limitations and to evaluate fine structural changes in bone microarchitecture with greater sensitivity and objectivity, Fractal Analysis (FA) has attracted increasing attention in the scientific literature [5].

FA, built on the concept of "fractals" first introduced by Mandelbrot (1967), is a non-invasive, objective method that mathematically examines the complex and self-similar architecture of bone trabeculae [1, 7, 9]. The Fractal Dimension (FD) derived from this analysis quantifies the morphological complexity, density, and porosity of bone tissue [7, 10]. Higher FD values generally indicate a more complex and well-organized bone structure [1, 7]; however, some studies have shown that FD may paradoxically decrease in cases where reactive bone returns to its normal density during healing [11, 12].

One of the major advantages of FA is its resistance to minor variations in projection angles and radiation doses, making it readily applicable to clinical radiographs [13, 14]. Nevertheless, a serious lack of methodological standardization persists in the literature, with FD calculations shown to be directly influenced by image resolution, ROI size, geometric orientation, box sizes, and preprocessing filters [10, 15–17]. These uncertainties represent a significant barrier to the consistency of scientific progress and the advancement of clinical applications.

The primary aim of this study was to systematically and rigorously identify the methodological parameters that maximize FA reliability — specifically ROI selection strategy, Gaussian filter sigma values, and box-counting algorithm choice — to present a reproducible standardized framework for 2D FA studies, and to strengthen clinical interpretability through effect sizes and diagnostic performance metrics. The secondary aim was to retrospectively evaluate how FA reflects clinical healing in periapical lesions whose healing status had been determined by PAI, in patients free of systemic conditions that adversely affect bone metabolism. To this end, non-healed patients with PAI ≥ 3 served not as a clinical subgroup for specificity testing but as a temporal stability reference to evaluate the consistency of FA parameters in the absence of expected biological change. While this approach does not constitute a strict biological negative control, it provides a methodologically valuable reference for testing the stability of the same protocol under conditions where change is not expected.

## Materıals and methods

### Study design, groups, and clinical non-healing reference group rationale

This retrospective study was conducted in accordance with the approval of the Karabük University Non-Interventional Clinical Research Ethics Committee (Date: 30.06.2022, Decision No: 1019). All procedures were carried out in compliance with the principles of the Declaration of Helsinki. The study data were compiled from the archival records of Karabük Oral and Dental Health Training and Research Hospital. All participants in the study provided informed consent.

The study was built upon two groups. The healed group (PAI < 3, *n* = 60) consisted of patients with confirmed bone healing after retreatment, in whom an increase in FD paralleling the decline in PAI score was biologically expected. The non-healed group (PAI ≥ 3, *n* = 35) comprised patients whose PAI score remained at baseline levels or who failed to demonstrate adequate healing. Because no appreciable trabecular reorganization was expected in this second group, FD values were predicted to remain stable throughout follow-up. The non-healed group was composed of real patients drawn from the same clinical context, the same imaging conditions, and the same FA protocol — rather than externally recruited healthy subjects. This feature renders the non-healed group methodologically valuable as a clinical non-healing reference group. This approach provides a valid methodological reference for testing the stability of the same protocol under conditions where change is not expected. The reference validity of this group rests on the assumption that no true bone microstructural change occurred over one year in patients with PAI ≥ 3; this assumption is supported by the PAI scores but cannot be confirmed with histological or 3D CBCT-based volumetric evidence. All retreatment procedures were performed by a single endodontic specialist.

**Inclusion criteria:** (i) endodontic retreatment performed on a mandibular molar tooth; (ii) baseline PAI score ≥ 3; (iii) baseline and 1-year follow-up periapical radiographs of adequate technical quality; (iv) absence of systemic conditions adversely affecting bone metabolism (diabetes, chronic kidney disease, primary hyperparathyroidism, osteoporosis, bisphosphonate use, etc.).

**Exclusion criteria:** technically inadequate images containing artifacts, elongation, foreshortening, or cone-cut; history of systemic disease or medication affecting bone metabolism.

A total of 220 digital periapical radiographs from 110 randomly selected patients were retrieved from the archive; the initial pool comprised 69 healed candidates and 41 non-healed candidates. Fifteen patients were excluded due to technical inadequacy (artifacts, elongation, foreshortening, or cone-cut): 9 from the healed candidates (69 → 60) and 6 from the non-healed candidates (41 → 35), yielding a final distribution of healed *n* = 60, non-healed *n* = 35, and total *n* = 95. The proportional distribution of excluded cases (healed: 9/69 = 13.0%; non-healed: 6/41 = 14.6%) was comparable to the final study group distribution, with no evidence of selective exclusion bias. The sample was restricted to mandibular molar teeth; this choice was motivated by the aim of enhancing radiographic standardization and minimizing projection geometry variation [18].

### Sample size and power analysis

Owing to the retrospective design, a prospective sample size calculation could not be performed. Nonetheless, the obtained effect sizes (Cohen's d = 1.07–1.22, healed group; d ≤ 0.07, non-healed group) and 95% confidence intervals (AUC = 0.91, 95% CI: 0.85–0.96) directly demonstrate that the sample was sufficient to detect clinically meaningful differences. The limitations of post-hoc power analysis are addressed in the Limitations section.

In the non-healed group of 35 patients, 7 ROI × 3 filter × 3 algorithm = 63 combinations were evaluated, with 35 paired measurements available for each combination. Because all 35 patients were assessed across all 63 combinations, the total number of measurements reached 35 × 63 = 2,205; however, since these measurements were derived from the same 35 patients, they do not fully satisfy the assumption of statistical independence. This should be regarded as evidence of empirical — not system-level — consistency; the consistently non-significant p-values (p > 0.05) and negligible effect sizes (d ≤ 0.07) obtained across all combinations empirically support the stability of the reference group, with the independence issue addressed within the mixed-effects GLM framework.

### Patient demographics

The demographic and clinical characteristics of the two groups are summarized in Table 1. The table also includes a "Baseline Pool" column to enhance transparency of the initial pool (*n* = 110) and the exclusion process. No statistically significant differences were found between the groups in terms of age and sex distribution (*p* > 0.05).

**Table 1:** Demographic and clinical characteristics of the study groups

Variable	Healed Group (*n* = 60) PAI < 3	Non-Healed Group (*n* = 35) PAI ≥ 3
Age — Mean ± SD (years)	38.4 ± 11.2	42.1 ± 13.6
Sex — Female/Male (n)	34/26	20/15
Baseline PAI score — Mean ± SD	3.8 ± 0.7	4.2 ± 0.9
Follow-up PAI score — Mean ± SD	1.8 ± 0.6	4.1 ± 0.8

*SD* Standard Deviation. From the initial archive pool (*n* = 110), 15 patients were excluded due to technical inadequacy (9 from healed candidates, 6 from non-healed candidates); this exclusion was proportionally distributed with no evidence of selective bias. Data in the table correspond to the final analysis groups (*n* = 60 and *n* = 35)

### Radiographic ımaging

Radiographic images were obtained using an Acteon Satelec X-mind intraoral X-ray unit at 70 kVp and 10 mA exposure settings. Standard size 2 phosphor plate sensors were used. Images were recorded in DICOM format at 8-bit grayscale depth with a pixel matrix of 1646 × 954. Given the sensor's active detection area (approximately 35 × 25 mm) and the image pixel matrix, the effective output resolution was calculated as approximately 1200 DPI (≈47 pixels/mm); the 300 DPI value in the DICOM header is a software-assigned metadata attribute and does not reflect the sensor's actual spatial resolution. Images were converted to lossless BMP format without modifying pixel values. Analyses were performed on standardized hardware (Intel® Core™ i3-5005U, 4 GB RAM, Lenovo B50-50).

All radiographs were taken using the bisecting angle technique. In the mandibular molar region, this technique is applied with an approximately 0–5° vertical angle, yielding a projection geometry that is nearly equivalent to the paralleling technique [18]. In the clinical setting from which this retrospective data was obtained, the sensor was held in place by the patient using finger pressure rather than a dedicated positioning device. While the absence of a positioning device may introduce minor inter-session projection angle variability, this limitation is mitigated by: (1) the near-parallel projection geometry of the bisecting angle technique in the mandibular molar region; (2) the established resistance of FA to minor projection angle variations [13, 14]; and (3) one-to-one ROI matching performed via the ROI Manager.

### Radiographic evaluation and healing criteria

Post-treatment healing status was evaluated according to the PAI system defined by Ørstavik et al. (1986) [8]. Baseline and 1-year follow-up radiographs were independently assessed by an oral and maxillofacial radiologist with 9 years of clinical experience and an endodontist. Inter-observer agreement was κ = 0.87 (95% CI: 0.81–0.93). In 12 cases where scores differed, a third independent expert made the decision. PAI < 3 was classified as healed and PAI ≥ 3 as non-healed.

### Fractal analysis protocol

**Fig. 1:**
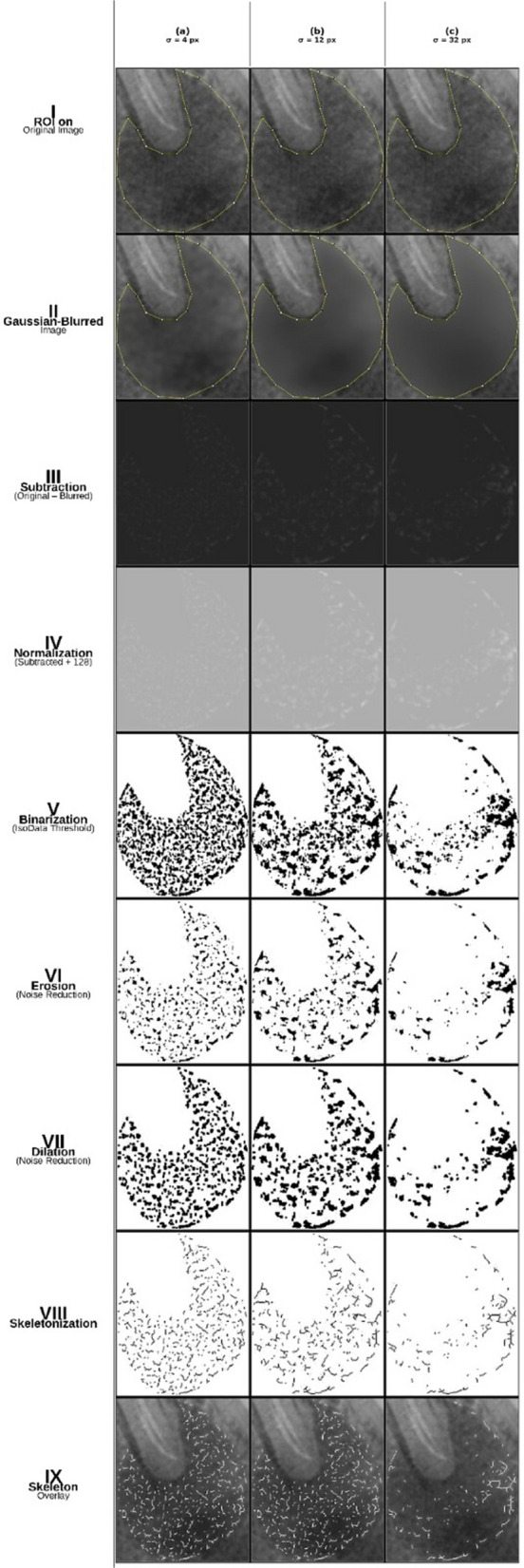
Image processing pipeline of fractal analysis applied to pre-treatment (pre-op) periapical radiographs of a representative case. Columns correspond to the three Gaussian sigma values tested: (a) σ = 4 px, (b) σ = 12 px, (c) σ = 32 px. Rows illustrate sequential processing steps: (I) ROI drawn on the original periapical radiograph; (II) Gaussian-blurred image; (III) Subtraction image (original − blurred); (IV) Normalization (subtracted + 128 Gy-level offset); (V) Binarization via IsoData automatic thresholding; (VI) Erosion; (VII) Dilation; (VIII) Single-pixel-width skeletonization; (IX) Skeleton overlay on the original radiograph

**Fig. 2:**
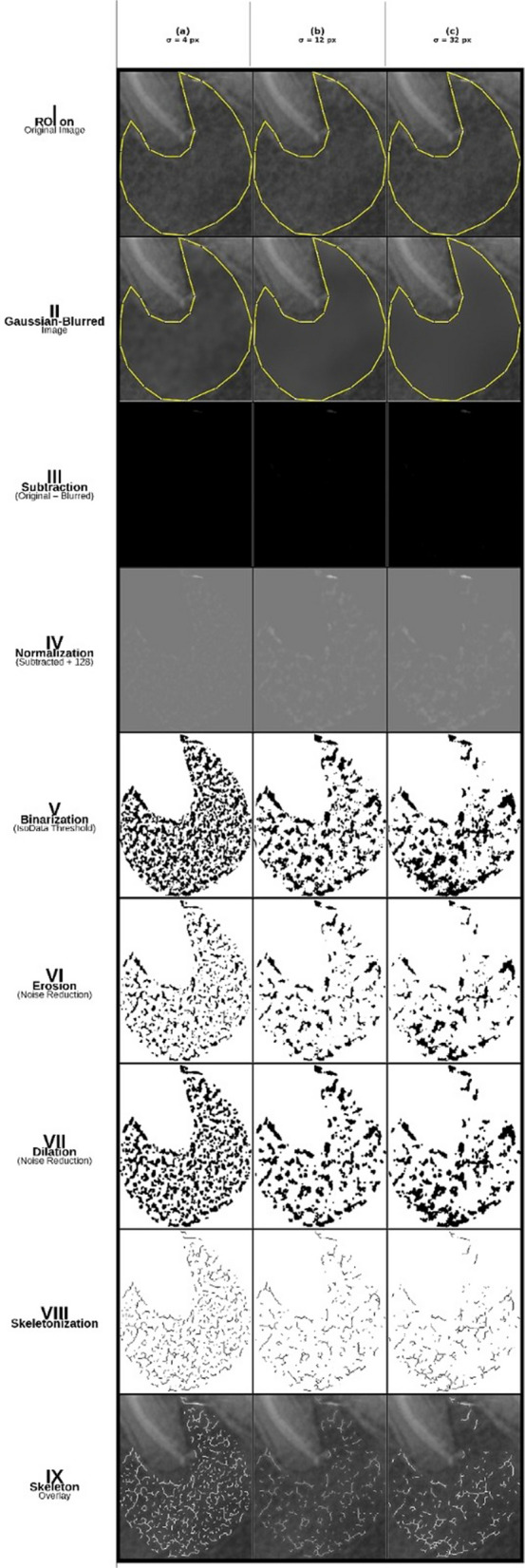
Image processing pipeline of fractal analysis applied to post-treatment (post-op, 1-year follow-up) periapical radiographs of the same case shown in Fig. 1. Post-treatment radiograph demonstrates increased trabecular complexity, reflected in higher binary pixel density and a denser skeleton, particularly under σ = 4 px (column a). This pattern is consistent with periapical bone healing confirmed by a reduction in PAI score

Fractal analysis was performed using Fiji/ImageJ v1.54f. ROIs were based on the method of White and Rudolph (1999) [19], with revisions to the ROI selection and image processing parameters as detailed below:

1. Gaussian Blurring: Three sigma values (σ = 4, 12, 32 pixels) were tested to eliminate low-frequency brightness gradients. The image processing steps for each sigma value are presented in detail on representative preoperative and postoperative radiographs (Figs. 1 and 2).

(2) Subtraction and Normalization: The blurred image was subtracted from the original; 128 was added to pixel values to shift negative values into the positive range.
(3) Binarization and Skeletonization: Images were converted to binary format using automatic IsoData thresholding. In keeping with the original White and Rudolph method — and contrary to some studies in the literature — the "invert" operation was deliberately omitted to prevent measuring intertrabecular spaces instead of bone trabeculae [19–21]. After noise reduction, single-pixel-width skeletonization was performed, and FD was calculated by the box-counting method. Three box-counting approaches were comparatively tested: (i) B64: classic grid scanning with a fixed series starting from a maximum box size of 64 pixels; (ii) B32: the same approach applied with a maximum box size of 32 pixels; (iii) BoneJ Auto (BA): an adaptive algorithm that automatically determines the starting and minimum box sizes, scaling factor, and grid translation count based on image dimensions, shifting the grid to the position yielding the minimum box count at each scale [22]. The superior ability of the BA algorithm to minimize quantization error compared with B64 and B32 is the fundamental source of its performance advantage.

### ROI selection protocol and observer reliability

**Fig. 3:**
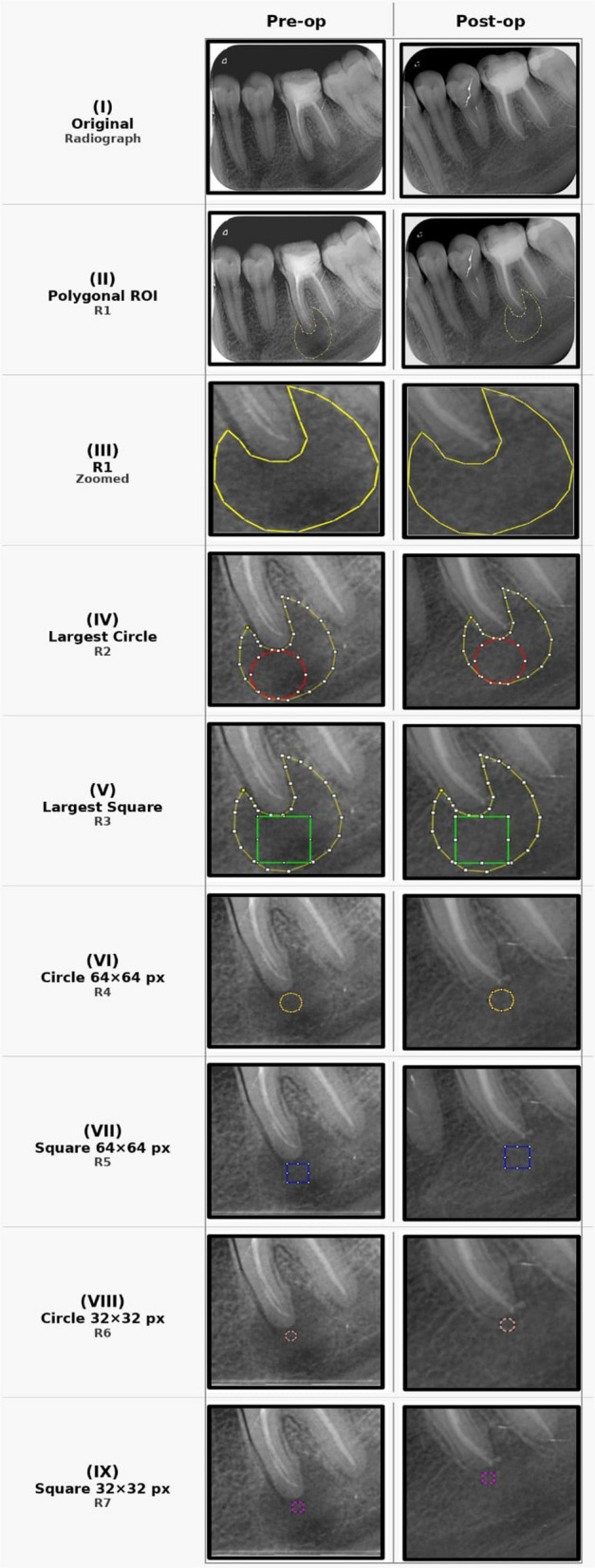
The seven ROI configurations evaluated in this study, illustrated on representative pre-treatment (Pre-op) and post-treatment (Post-op, 1-year) periapical radiographs. Each row shows one ROI type applied to the same tooth; left column = pre-op, right column = post-op. (1) Original radiograph without ROI overlay. (2) Manually traced polygonal ROI (ROI-1). (3) Enlarged view of ROI-1. (4) Largest circle inscribed within ROI-1 (ROI-2). (5) Largest square inscribed within ROI-1 (ROI-3). (6) Fixed circular ROI, 64 × 64 px, apex-centered (ROI-4). (7) Fixed square ROI, 64 × 64 px, apex-centered (ROI-5). (8) Fixed circular ROI, 32 × 32 px, apex-centered (ROI-6). (9) Fixed square ROI, 32 × 32 px, apex-centered (ROI-7). Note: rows (1) and (3) are reference views, not separate ROI types. ROI-1–ROI-3: lesion-focused ROIs; ROI-4–ROI-7: apex-centered fixed-size ROIs

Seven ROI strategies were employed to evaluate bone healing across different anatomical regions (Table 2, Fig. 3). All ROI types are consistently designated as ROI-1 through ROI-7 throughout tables and text. The boundaries of the polygonal ROI (ROI-1) were drawn by two independent observers based on the following criteria: (i) the outer margin of the radiolucent area, (ii) the boundary where lamina dura integrity was lost, and (iii) the outer contour following the tooth surface without encompassing the root or apex. To determine intra-observer and inter-observer reliability, ROI delineations and FD measurements were repeated on 20 randomly selected cases from the total sample. Inter-observer reliability was tested through independent measurements by a maxillofacial radiologist with 9 years of experience and an endodontist; intra-observer reliability was assessed by the first observer re-evaluating the same images at a two-week interval. A two-way mixed-effects model for absolute agreement with single rater/measurement (ICC 3,1) was used for statistical evaluation. Inter-observer ICC was 0.91 (95% CI: 0.85–0.95) and intra-observer ICC was 0.94 (95% CI: 0.89–0.97), indicating "excellent" measurement reproducibility.

**Table 2:** Definitions, dimensions, and anatomical localization criteria of the ROI configurations used for fractal analysis

ROI	Geometry/Size	Definition
ROI-1	Manual Polygonal	Manually delineated area encompassing the lesion boundaries (lamina dura loss and radiolucent area) on the baseline radiograph, excluding dental tissue
ROI-2	Largest Inscribed Circle	The largest circular area that fits within the ROI-1 polygon, representing the center of the lesion
ROI-3	Largest Inscribed Square	The largest square area that fits within the ROI-1 polygon, representing the center of the lesion
ROI-4	Circle with 64 px diameter	Fixed circular area positioned immediately apical to the root apex (excluding dental tissue), using the root apex as a reference point
ROI-5	64 × 64 px Square	Fixed square area positioned immediately apical to the root apex, using the root apex as a reference point
ROI-6	Circle with 32 px diameter	Smaller-scale fixed circular area positioned immediately apical to the root apex, using the root apex as a reference point
ROI-7	32 × 32 px Square	Smaller-scale fixed square area positioned immediately apical to the root apex, using the root apex as a reference point

### Statistical analysis

Statistical analyses were conducted using Minitab® 21 (Minitab LLC, State College, PA, USA). Normal distribution was verified by the Anderson–Darling test, and homogeneity of variance by Levene's test. The significance level was set at p < 0.05 for all tests.

As the primary analysis, a mixed-effects GLM was fitted using Minitab® 21's "Fit Mixed Effects Model" procedure: patient ID was defined as a random effect, while ROI type, filter, and algorithm were fixed effects. This model evaluated all 63 parameter combinations (7 ROI × 3 filter × 3 algorithm) within a single framework, accounting for the within-patient correlation of repeated measurements. Defining patient ID as a random effect explicitly accounts for the correlation structure among repeated measurements derived from the same patient — a standard statistical solution to pseudoreplication. Fixed effects are estimated by controlling only for patient-level variance. Accordingly, the 11,970 individual measurements (5,985 data pairs) are not treated as independent observations but as data derived from 95 patients with within-patient correlation modeled explicitly. The mean differences and Cohen's d values reported in the tables reflect the marginal (weighted mean) estimates of this model. Cohen's d values were calculated using the formula appropriate for paired designs: d = Δmean/SD_diff; the corresponding SD_diff values are presented in Table 4. The data structure of the study was as follows: 3,780 paired measurements across 63 parameter combinations for each patient in the healed group (*n* = 60), and 2,205 paired measurements in the non-healing reference group (*n* = 35), for a total of 5,985 data pairs (11,970 individual FD values). This large data volume was managed within the mixed-effects GLM framework by defining patient ID as a random effect, thereby controlling the clustering effect of measurements from the same patient and minimizing the risk of type I error.

As a confirmatory analysis, paired t-tests were applied at the region × combination level. Benjamini–Hochberg FDR correction (q < 0.05) was applied to control for the multiple comparison problem across 63 combinations. For diagnostic performance, ROC curve and AUC calculations were performed on data from the ROI-1 + BA + σ4 combination using the software's logistic regression module (AUC = 0.91, 95% CI: 0.85–0.96). The selection of this combination for ROC analysis was not post hoc: the combination was determined based on predefined criteria (effect size magnitude + significance consistency) as the intersection of the algorithm showing the highest marginal effect size in the GLM analysis (BA, d = 1.07), the filter with the highest discriminatory power (σ4, d = 1.12), and the ROI type with the strongest anatomical validity (ROI-1, polygonal area faithful to lesion boundaries). The selection of ROI-1 rested not only on its statistical performance but also on its capacity to most completely represent true biological change through its polygonal geometry that encompasses the entire lesion while adhering to anatomical boundaries. No external validation cohort was available in this study; therefore, the diagnostic performance findings should be considered exploratory and interpreted with caution until confirmed in an independent sample. The limitations of ROC analysis applied to a single combination and the need for cross-validation approaches in future studies are addressed in the Limitations section.

## Results

### Healed group — overall assessment of FD change

Mixed-effects GLM analysis revealed that all main factors (ROI type, algorithm, and filter) had a statistically significant effect on FD change in the healed group (p < 0.001). After Benjamini–Hochberg FDR correction, all findings for ROI-1 through ROI-5 remained below the q < 0.05 threshold; ROI-1, encompassing the largest anatomical area, exhibited the highest marginal difference. Among algorithms, BoneJ Auto (BA) clearly distinguished itself from the other approaches with d = 1.07, well exceeding the "large" effect threshold (d ≥ 0.80). Among Gaussian filters, σ = 4 px achieved the highest discriminatory power with d = 1.12. ROI-6 and ROI-7 maintained statistical non-significance across all combinations both before and after correction (p > 0.05); these small fixed-size ROIs carry no clinical informational value for FA (Table 3).

**Table 3:** Marginal effect assessment of FD change by factor in the healed group (Mixed-Effects GLM)

Category	Subcategory	Mean Diff	Cohen's d	*p*-Value	*q*-Value (BH)
**Region (ROI Type)**	ROI-1	+ 0.121	0.82	< 0.001	< 0.05
	ROI-2	+ 0.115	0.78	< 0.001	< 0.05
	ROI-3	+ 0.108	0.74	< 0.001	< 0.05
	ROI-4	+ 0.098	0.64	0.002	< 0.05
	ROI-5	+ 0.091	0.60	0.004	< 0.05
	ROI-6	+ 0.035	0.22	0.185	> 0.05
	ROI-7	+ 0.031	0.20	0.241	> 0.05
**Algorithm**	BA (BoneJ Auto)	** + 0.185**	**1.07**	< 0.001	< 0.05
	B64 (Fixed 64 px)	+ 0.118	0.80	< 0.001	< 0.05
	B32 (Fixed 32 px)	+ 0.108	0.73	< 0.001	< 0.05
**Gaussian Filter (σ)**	σ = 4 px	** + 0.155**	**1.12**	< 0.001	< 0.05
	σ = 12 px	** + 0.141**	**1.01**	< 0.001	< 0.05
	σ = 32 px	+ 0.114	0.77	< 0.001	< 0.05

Values reflect weighted mean estimates across all region or parameter levels. q-values were computed according to Benjamini–Hochberg FDR correction. Bold d values: ≥ 1.0. Gaussian filter abbreviations (σ4, σ12, σ32) are independent of ROI type designations (ROI-1–ROI-7)

Note: The abbreviations σ4, σ12, σ32 in the "Gaussian Filter" rows of Table 3 refer to the sigma parameter of the Gaussian kernel and are independent of the ROI type designations ROI-1 through ROI-7

### Healed group — region-specific optimal combinations

Combination comparisons by ROI in the healed group, including effect sizes and SD_diff values, are presented in Table 4. For each ROI, the upper row represents the combination with the highest effect size and the lower row the one with the lowest. Regions can be evaluated in three subcategories based on their diagnostic performance. (a) Highly discriminative regions (ROI-1–ROI-3): the BA + σ4 combination produced "very large" effect sizes in the range d = 1.18–1.22, clearly demonstrating the diagnostic superiority of lesion-focused large ROIs. (b) Partially significant regions (ROI-4–ROI-5): strong significance was maintained with the BA + σ4 combination (p < 0.001, d = 1.05–1.07), but the lowest-performing combinations (B32 + σ32) failed to reach the significance threshold (p > 0.05) and the effect size dropped to negligible levels (d = 0.30–0.32). (c) Regions without clinical informational value (ROI-6–ROI-7): all combinations yielded p > 0.05 and d < 0.25; these small, fixed-size square ROIs fall below the minimum area required for FA to produce stable results.

**Table 4:** Combination comparisons by ROI in the healed group (paired t-test)

Region	Combination	Mean Increase	Cohen's d	SD_diff	p-Value
**ROI-1**	**BA + σ4**	** + 0.218**	**1.20**	0.182	< 0.001
	B32 + σ32	+ 0.075	0.41	0.183	0.028
**ROI-2**	**BA + σ4**	** + 0.215**	**1.18**	0.182	< 0.001
	B32 + σ32	+ 0.063	0.35	0.180	0.049
**ROI-3**	**BA + σ4**	** + 0.221**	**1.22**	0.181	< 0.001
	B32 + σ32	+ 0.069	0.38	0.182	0.039
**ROI-4**	**BA + σ4**	** + 0.191**	**1.05**	0.182	< 0.001
	B32 + σ32	+ 0.058	0.32	0.181	0.061 (n.s.)
**ROI-5**	**BA + σ4**	** + 0.195**	**1.07**	0.182	< 0.001
	B32 + σ32	+ 0.054	0.30	0.180	0.075 (n.s.)
**ROI-6**	B64 + σ4	+ 0.049	0.22	0.222	0.112 (n.s.)
	B32 + σ32	+ 0.011	0.06	0.183	0.412 (n.s.)
**ROI-7**	B64 + σ4	+ 0.047	0.21	0.224	0.121 (n.s.)
	B32 + σ32	+ 0.009	0.05	0.180	0.498 (n.s.)

For each ROI, the upper row represents the combination with the highest effect size and the lower row the lowest. The SD_diff column allows independent verification of Cohen's d values (d = Δmean/SD_diff). n.s.: not statistically significant (*p* > 0.05)

The strongest diagnostic performance was achieved with the ROI-1 + BA + σ4 combination: ROC analysis yielded an exploratory AUC = 0.91 (95% CI: 0.85–0.96), which should be interpreted with caution given the absence of external validation, cross-validation, or a training/test split. The numerical differences between tables do not represent a methodological inconsistency: the weighted mean values in Table 3 (e.g., + 0.185 for BA) reflect the average across all ROIs (including the low-value ROI-6 and ROI-7), whereas the values in Table 4 are paired t-test results specific to individual ROI–combination pairs. SD_diff values are presented in Table 4 to enable independent verification of Cohen's d calculations (d = Δmean/SD_diff).

### Non-healing reference group — clinical reference validation

In the clinical non-healing reference group (PAI ≥ 3, *n* = 35), no statistically significant FD change was detected in any ROI or combination over the 1-year follow-up period (all regions p > 0.05; all combinations d ≤ 0.07). The fact that all effect sizes remained at negligible levels confirms the temporal stability of the measurement system within a clinically non-healed cohort — a finding that provides methodological evidence that the protocol does not generate false-positive signals in the presence of persistent lesions under the conditions of this study, while noting that this cannot be interpreted as formal diagnostic specificity given the PAI-only classification. Notably, the BA + σ4 combination — which provided the highest discriminatory power in the healed group — exhibited complete temporal stability in the non-healing reference group (ROI-1: d = 0.05, p = 0.742), demonstrating that high sensitivity and temporal stability in the absence of change converge within the same parameter set (Table 5).

**Table 5:** FD change analysis in the non-healing reference group (PAI ≥ 3, *n* = 35)

ROI	Best Combination	Mean Diff	Cohen's d	*p*-Value
ROI-1	BA + σ4	− 0.008	0.05	0.742
ROI-2	BA + σ4	− 0.011	0.07	0.681
ROI-3	BA + σ4	+ 0.004	0.03	0.891
ROI-4	BA + σ4	+ 0.006	0.04	0.832
ROI-5	BA + σ4	− 0.009	0.06	0.714
ROI-6	B64 + σ4 (best)	+ 0.003	0.02	0.924
ROI-7	B64 + σ4 (best)	− 0.002	0.01	0.963

*clinical non-healing reference validation. All combinations p* > *0.05 and d* ≤ *0.07. For ROI-6 and ROI-7, data for the combination with the highest effect size are presented*

## Dıscussıon

This study demonstrated, through two complementary lines of evidence, that FA can detect periapical bone healing after non-surgical endodontic retreatment. While FA parameters detected a significant FD increase with large effect sizes (Cohen's d > 1.0) in the healed group, the non-healing reference group confirmed that the same protocol remained temporally stable, with no statistically significant or clinically meaningful FD change (d ≤ 0.07) in the absence of true biological change. The convergence of these findings indicates that an optimized FA protocol carries high clinical value in terms of both sensitivity (capturing healing) and temporal stability in the absence of change — while noting that the latter cannot be equated with formal diagnostic specificity given the PAI-only classification used in this study.

### Methodological Value and Limitations of the Clinical Non-Healing Reference Group Approach

Many FA studies compare the healing group with externally recruited healthy subjects or contralateral teeth [2, 14, 16]. One of the original contributions of the present study is the utilization of non-healed patients from the same clinical context as a clinical non-healing reference group. Similar designs supporting this methodological approach exist in the literature: Oliveira et al. (2025) [23] advocated the use of reference data obtained from the same clinical context for comparative evaluation of FA parameters between healed and non-healed groups; Huang et al. (2013) [12] and Yu et al. (2009) [11] used FA stability in non-healed or active lesions as a methodological reference. For this approach to be considered robust, however, the assumption that no true bone microstructural change occurred over one year in patients with PAI ≥ 3 must hold. This assumption is supported by PAI scores and reinforced by clinical outcome stability, demographic equivalence, and a single-center standard imaging protocol; nevertheless, it cannot be confirmed with histological or 3D CBCT evidence. For this reason, the non-healed group is characterized as a "clinical non-healing reference group" rather than a "strong biological negative control."

### The critical role of ROI selection strategy: lesion-focused vs. size-focused approaches

The dramatic effect of ROI selection strategy on FD results is among the most important findings of our study. Large polygonal or geometric ROIs encompassing the entire lesion (ROI-1, ROI-2, ROI-3) detected treatment-related FD changes with high sensitivity, while small standard ROIs positioned at a fixed distance from the root apex (ROI-6, ROI-7) produced statistically non-significant results. The failure of small 32 × 32 pixel ROIs is directly related to an inadequate sampling volume that cannot represent the microarchitecture of heterogeneous trabecular tissue — a situation that can be explained by the classical concept of "sampling bias" [15, 24]. Indeed, other medical fractal studies such as osteoporosis screening [25] and bone turnover monitoring [24] have also emphasized that the "largest selectable ROI" strategy is essential for microstructural representativeness.

The "apex-centered/fixed-distance" ROI placement widely adopted for ROI standardization in the endodontic literature [1, 26] offers reproducibility but carries the risk of ignoring the asymmetric biological behavior of the pathology. The fact that ROI-1 (lesion-focused polygonal) exhibited the highest discriminatory power validates the approach of adhering to biological boundaries rather than geometric standardization in ROI selection. This finding supports the pioneering work of Oczeretko et al. [27] on irregularly shaped ROIs and the recommendation of Cavalcante et al. [25] to "select the largest possible area." An additional critical reason for avoiding square ROIs is the Direction of Region of Interest Selection (DROIS) effect: square ROIs are sensitive to minor angular and rotational differences in the radiograph and carry the risk of placement bias [15]. In contrast, circular and polygonal ROIs provide direction-independent "geometric stability," reducing susceptibility to DROIS-related placement bias [15].

### Scale-space optimization for gaussian sigma selection and resolution of technical ınconsistencies

The Gaussian sigma (σ) value, one of the most critical parameters in the image processing stage, was grounded in a rational and mathematical basis that transcends dogmatic approaches in the literature. The σ = 35 and kernel size = 33 × 33 parameters proposed by White and Rudolph [19] in 1999 were compatible with the software architecture of that era (NIH Image) and represented a valid configuration for scanned film-based 600 DPI images. However, directly transferring these historically inherited parameters to current software platforms (ImageJ/Fiji) without technological adaptation to the high resolution of modern digital detectors constitutes a core methodological problem. It should be noted that in modern versions of ImageJ (v1.41 and later), the Gaussian Blur filter no longer requests a separate "kernel size" input from the user; only the σ (sigma) parameter can be calibrated, with the software automatically computing the kernel size in the background using the formula 6σ + 1 (or 2 × ⌈3σ⌉ + 1) [28, 29]. Under this formula, σ = 35 requires an enormous 211 × 211 pixel filter kernel — a size that vastly exceeds the boundaries of the small standard ROIs used in our study (32 × 32 or 64 × 64) and leads to the erasure of trabecular information through "edge artifacts" [30, 31]. A noteworthy observation is that although many contemporary researchers state they use ImageJ, they continue to report a manually entered kernel value (e.g., 33 × 33) when describing Gaussian filter parameters — in a manner inconsistent with the software's current architecture — suggesting that the computational origin and current operation of this parameter are not fully understood.

To overcome this technical obstacle, filter values in our study were determined within the framework of "Scale-Space Theory" (Lindeberg, 1994) [32] using logarithmic sampling and geometric progression principles. Accordingly, σ_mi*n* = 4 (minimal intervention) and σ_max = 32 (2^5^, aggressive background elimination) were selected as endpoints; the intermediate value was calculated by the geometric mean method (σ_mid = √(4 × 32) ≈ 11.3 pixels), which provides perfect symmetry on a logarithmic scale [33]. Rounded to 12 pixels for practical convenience, this intermediate value also aligns with the literature average (10–12 pixels) [5, 31]. The set of 4, 12, and 32 pixels determined by geometric progression offers a 3-octave (2^3^) wide dynamic range, enabling the analysis of bone structures at different spatial scales [4, 34]. The physical basis for the most sensitive results being obtained with σ = 4 is directly related to the sensor's actual output resolution of approximately 1200 DPI (≈47 pixels/mm): σ = 4 pixels corresponds to a physical blurring of 4/47 ≈ 0.085 mm at this resolution. This value falls below the average trabecular thickness (0.1–0.3 mm [35]) and provides a mathematically justified lower bound consistent with the minimal preprocessing principle [36].

Another critical issue directly linked to filter parameters is the correspondence between maximum box size and ROI size. The mathematical validity of the box-counting algorithm depends on the reliability of the log–log regression line (scaling region). As a standard rule, the largest box size used must not exceed 50% of the ROI dimension [37, 38]. The static B64 and B32 lists used in our study violate this rule when applied to small ROIs such as ROI-6 and ROI-7 (32 × 32), producing insufficient data points for regression and yielding mathematically invalid FD values [15, 37].

### Binarization and algorithm superiority: the mathematical robustness of the ba algorithm

During the binarization stage, the "invert" operation was deliberately avoided — contrary to some studies in the literature [39–41]. As Silva et al. [21] pointed out, the invert operation causes the algorithm to measure intertrabecular spaces (bone marrow) instead of bone trabeculae, thereby rendering FD results artificial. The superior performance of the BoneJ Auto (BA) algorithm over fixed geometric series (B64, B32) in the computation stage stems not merely from its "automatic" nature but from its "multigrid shifting" capability. The BA algorithm does not keep the grid at a fixed position but shifts it across different starting coordinates on the image, finding the minimum box count at each scale — a feature that minimizes "quantization noise" [22, 42]. Furthermore, BA dynamically generates the mathematically optimal box series for each ROI size without violating the 50% image size rule [37, 43]. In contrast, static box lists such as B32 and B64 violate this rule in small ROIs like ROI-6 and ROI-7 (32 × 32), producing insufficient data points for regression and generating mathematically invalid FD values [15, 37]. The sensitivity of our proposed integrated protocol (minimal σ = 4 filter and BA algorithm) has been biologically validated by recent studies that detected rapid bone turnover even during the pubertal growth spurt [24].

### Clinical Significance and Contribution to Decision-Making: The "Radiographic Gray Zone" and an Additional Quantitative Measure Complementary to PAI

An exploratory AUC of 0.91 (95% CI: 0.85–0.96) — derived from a single parameter combination without external validation, cross-validation, or a training/test split, and therefore subject to overfitting and optimism bias — indicates that the ROI-1 + BA + σ4 combination has the potential for near-excellent discriminatory performance in distinguishing healed from non-healed patients, pending independent confirmation. The clinical implication of this finding is directly related to the "radiographic gray zone" that forms during the transition between a PAI score of 3 (lesion present) and 2 (return to normal): the observer-dependent, ordinal structure of PAI predisposes to subjective assessment errors during this critical transition phase. The quantitative rate of FD increase offers a higher signal-to-noise ratio compared to the subjectivity of the PAI system; the effect size of d = 1.07 demonstrates that the FD increase measured with the BA + σ4 combination provides superior signal clarity over PAI change at the patient level. From this perspective, fractal dimension may serve as an additional quantitative measure complementary to PAI, which may provide supplementary objective evidence in cases of diagnostic uncertainty — such as the decision to continue conservative follow-up versus proceeding to surgical retreatment. While an FD increase is heavily correlated with clinical healing, bone remodeling is a multifaceted biological process that may involve complex phases such as sclerosis, fibrosis, corticalization, reactive bone formation, or heterogeneous trabecular changes. Therefore, FD values are best interpreted as reflections of these dynamic microarchitectural alterations rather than a singular, definitive state of healing. This finding positions FA not as a screening tool but as a quantitative confirmation tool complementary to PAI.

### Limitations and methodological consistency

Despite the superimposition limitation inherent to 2D radiographs [13, 14], the one-to-one pre-op/post-op matching achieved through the ROI Manager enhanced the objectivity of the study.

(i) Although the retrospective design carries the risk of projection differences, the nearly parallel projection geometry afforded by the bisecting angle technique in the mandibular molar region [18] and the established resistance of FA to minor angle variations [13, 14] minimize this risk; sensor positioning via finger pressure rather than a dedicated device is an additional source of minor inter-session variability; (ii) the anatomical superimposition constraint of 2D images represents a fundamental limitation: without 3D CBCT confirmation, lesion volume reduction, cortical healing, and three-dimensional trabecular regeneration cannot be reliably assessed, and the biological interpretation of FD changes in this study is therefore constrained to the 2D radiographic domain — CBCT-based validation is a necessary direction for future research; (iii) post-hoc power analysis does not fully substitute for a prospective design; (iv) while one year of follow-up represents an important early phase of the healing process, longer time intervals are needed for long-term changes; (v) the manual polygonal ROI approach (ROI-1) is the basis of the proposed optimized protocol and yielded excellent ICC values (inter-observer ICC = 0.91; intra-observer ICC = 0.94); however, these values do not eliminate inherent subjectivity in boundary delineation, which may vary depending on observer interpretation, radiographic contrast, and cortical overlap — particularly in cases with indistinct lesion margins; future studies may benefit from semi-automated or AI-assisted lesion boundary detection to reduce observer dependence; (vi) healing status was determined solely by PAI — histological or 3D CBCT-based confirmation was not performed — and the temporal stability findings in the non-healing reference group therefore cannot be equated with formal diagnostic specificity; (vii) formal statistical power at the combination level is limited in the non-healing group (*n* = 35); while empirical consistency findings (d ≤ 0.07 across all combinations) support this limitation, confirmation with larger samples is recommended; (viii) ROC analysis was applied to a single combination without external validation, cross-validation, or a training/test split — the reported AUC of 0.91 is therefore an exploratory estimate subject to overfitting and optimism bias, and should be confirmed in an independent sample using appropriate validation strategies.

## Conclusıon

This study confirmed, through two complementary lines of evidence, that FA can detect periapical bone healing after endodontic retreatment with sensitivity and temporal stability. In the healed group, FD showed a significant increase with large effect sizes (d = 1.07, exploratory AUC = 0.91 [ROI-1, BA + σ4] — pending independent validation), while the non-healing reference group demonstrated that the same parameters remained temporally stable and free of statistically significant or clinically meaningful FD change in the absence of true biological change (d ≤ 0.07 across all combinations). The study's most significant methodological contribution is the systematic demonstration that FD measurement reliability is deeply dependent on technical parameters, and that the conventional σ = 35 parameter represents a historically inherited parameter that may not be directly transferable to modern digital imaging platforms. The biological interpretation of FD changes is constrained to the 2D radiographic domain; without 3D CBCT confirmation, lesion volume reduction and three-dimensional trabecular regeneration cannot be reliably verified, and CBCT-based validation remains a necessary direction for future research. When all findings are considered together, a three-component "Optimized Standard Protocol" is recommended for 2D fractal analysis studies: (i) Representativeness — a lesion-focused, maximally inclusive circular/polygonal ROI (ROI-1 or ROI-2) that prevents sampling bias and reduces susceptibility to DROIS-related placement bias; (ii) Sensitivity — a low sigma filter (σ = 4) compatible with the sensor's ~ 1200 DPI output resolution, delivering physical blurring below trabecular thickness; (iii) Mathematical Accuracy — a BoneJ Auto (BA) algorithm that minimizes quantization noise through multigrid shifting. This triple combination stands out as a standardized methodological framework offering high signal-to-noise ratio, theoretical consistency, and biological accuracy for endodontic healing monitoring.

## Data Availability

The datasets generated and/or analyzed during the current study are not publicly available due to patient privacy and institutional data protection policies but are available from the corresponding author on reasonable request.
